# Association between homocysteine levels in acute stroke and poststroke depression: A systematic review and meta‐analysis

**DOI:** 10.1002/brb3.2626

**Published:** 2022-05-23

**Authors:** Yupei Chen, Hua Zou, Meidi Peng, Yan Chen

**Affiliations:** ^1^ School of Medicine (School of Nursing) Nantong University Nantong Jiangsu China; ^2^ Department of Neurology Nantong Third People's Hospital, Nantong University Nantong Jiangsu China

**Keywords:** depression, meta, poststroke depression, stroke, homocysteine

## Abstract

**Background:**

Homocysteine (Hcy) has been confirmed to be associated with depression, but its relationship with poststroke depression (PSD) remains controversial. So far, there is no meta‐analysis of the correlation between Hcy level in acute stroke and PSD.

**Methods:**

A systematic search of a sub‐database of studies reporting the level of Hcy in the acute phase of ischemic stroke and PSD as of November 2021 was performed. Data extraction was performed strictly according to the inclusion and exclusion criteria. All data were analyzed using STATA 11.0. The standardized root mean square difference (SMD) and 95% confidence interval (CI) were used to compare continuous variables.

**Results:**

A total of 11 studies were included in this study, including 2789 participants. The results of this meta‐analysis showed that admission the levels of Hcy were significantly higher in PSD survivors, compared to non‐PSD survivors (SMD = 0.37, 95%CI = 0.07–0.66, *P* ＜ .001). Subgroup analysis showed that survivors with PSD diagnosed more than 3 months after stroke had significantly different the levels of from non‐PSD survivors (6 months: SMD = 0.61, 95%CI = 0.40–0.82, 9 months: SMD = 1.00, 95%CI = 0.59–1.41).

**Conclusion:**

The level of Hcy in the acute phase of ischemic stroke is a risk factor for PSD.

## Graphical abstract

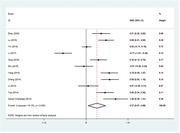

## 1 INTRODUCTION

Stroke is a vital cause of death, disability, and reduced life expectancy worldwide (GBD 2016 Disease and Injury Incidence and Prevalence Collaborators, 2017). Poststroke depression (PSD) is the most common noncognitive neuropsychiatric complication after stroke, and about 1/3 of stroke survivors suffer from PSD (Medeiros et al., 2020; Mitchell et al., 2017).

It is mainly manifested in mental symptoms such as depression, decreased interest, fatigue, or atypical and hidden symptoms such as physical pain, numbness, and even denial of disease (Tateno et al., 2002). PSD not only affected the rehabilitation (Paolucci, 2017), but also had connection with increased risk of all‐cause mortality significantly (Cai et al., 2018). The risk factors of PSD are complex and diverse, for instance, age, sleep time, education level, race (Tateno et al., 2002), stroke location and stroke severity (Shi et al., 2017). Currently, the pathogenesis of PSD has not unified explanation clearly. There are many hypotheses, including monoaminergic hypothesis (Hama et al., 2017; Terroni et al., 2011), hypothalamic–pituitary–adrenal (HPA) axis imbalance, glutamate‐mediated excitotoxicity, inflammatory response, and so on (Hama et al., 2017; Loubinoux et al., 2012).

Hcy, as a sulfur‐containing amino acid, may cause abnormalities of central monoamine neurotransmitters and HPA axis by changing neurotransmitter (Bryer et al., 1992), to aggravate the degree of depression. A review (Folstein et al., 2007) in 2007 showed strong published evidence of the association between Hcy levels and depression, vascular disease. The result of a subsequent large population study (Nabi et al., 2013) based on 3992 cases also showed that elevated levels of Hcy were associated with depression. Additionally, Hcy is considered as an evaluation index of stroke improvement (Zhang et al., 2021; Zhang et al., 2020). However, the effect of Hcy levels in the acute stage of stroke on PSD remains controversial (Unal et al., 2013; Zheng et al., 2019). Therefore, we systematically reviewed the evidence and conducted a meta‐analysis to explore whether Hcy levels in the acute phase of stroke have an association with PSD.

## 2 MATERIALS AND METHODS

The meta‐analysis was performed according to the Preferred Reporting Items for Systematic Reviews and Meta Analysis guidelines (PRISMA) (Page et al., 2021). Ethical approval was unnecessary in this study because this paper was based on the statistical analysis of previous articles.

### 2.1 Search strategy

The systematic review was conducted by using PubMed, Web of Science, EMBASE and the Cochrane Library. The relevant studies published until November 2021 were searched from above databases. The following searching strategy was used: (“plasma homocysteine” OR “homocysteine” OR “Hcy” OR “plasma Hcy”) AND (“post‐stroke of depression” OR “depression after stroke” OR “post‐stroke depression” OR “poststroke depression” OR “post stroke depression” OR “PSD”). Among the studies with overlapping data published by the same author, only the latest or complete studies were included in this meta‐analysis.

### 2.2 Selection criteria

Two researchers (CYP and ZH) worked independently to assess the eligibility of literature. The following criteria were used to identify eligible studies that had investigated the association between Hcy levels and PSD:

(1) Design: cohort or case control study; (2) participants: meet the corresponding diagnostic criteria of stroke in the World Health Organization standard, and PSD was assessed by professional scale test; (3) language: English ; (4) studies: Hcy levels at stroke onset must be provided; and (5) data could be extracted.

The exclusion criteria were as follows: (1) repetitive published literature; (2) abstracts, case reports, press releases, proceedings, letters, reviews, and meta‐analysis; (3) incomplete outcome data; and (4) score less than 5 stars on the Newcastle–Ottawa scale (NOS). Disagreements were figured out by consulting the third researcher (PMD).

### 2.3 Data extraction

All the included articles were independently read by two researchers trained in systematic review (CYP and ZH) and screened in strict accordance with the inclusion criteria. The first author, publication time, sample size, country of origin, diagnosis of stroke and PSD, time points when PSD was assessed, Hcy levels (mean ± standard), and other elements will be extracted from the included articles according to the standard format. When the data on the Hcy levels were reported as medians and interquartile ranges, we referenced Hozo et al.’s (2005) methods which simple and elementary inequalities and approximations in order to estimate the mean and standard deviation (SD) of the sample for further analysis. The test selection details are shown in the flow chart that complied with the Preferred Reporting Items for a systematic review and meta‐analysis.

### 2.4 Literature quality assessment

Literature quality evaluation two researchers independently used the Newcastle–Ottawa scale (NOS) to evaluate the quality of the included cohort or case control studies. The score of NOS is represented by stars, mainly including Selection (4 items), Comparability (1 item) Outcome (3 items), a total of eight items. Each item is represented by 1 star when appropriate. The comparability module can obtain up to 2 stars, and the full score is 9 stars. Generally, those with literature quality ≤4 stars are low‐quality studies, and those with 5–9 stars are high‐quality studies. The scale has been widely used in the quality evaluation of cohort or case control studies. Any differences will be resolved through group discussion.

### 2.5 Data analysis

Statistical analyses were carried out by using STATA 11.0. Judge the heterogeneity between different studies via *I* ^2^ statistic (Higgins et al., 2003). When *I* ^2^ ≤50%, it indicates that the heterogeneity between studies is low, and the fixed‐effects model should be used for analysis. When *I* ^2 ^> 50%, it indicates that there is high heterogeneity among studies and a random‐effects model was used. Sensitivity analysis and subgroup analysis are used to find the source of heterogeneity. Publication bias was assessed by funnel plot and Egger test. SMD and 95% CI were used to describe continuous data and to estimate the relationship between Hcy levels and the risk of PSD in this analysis. The results were represented by forest plots.

## 3 RESULTS

### 3.1 Literature search

**FIGURE 1:**
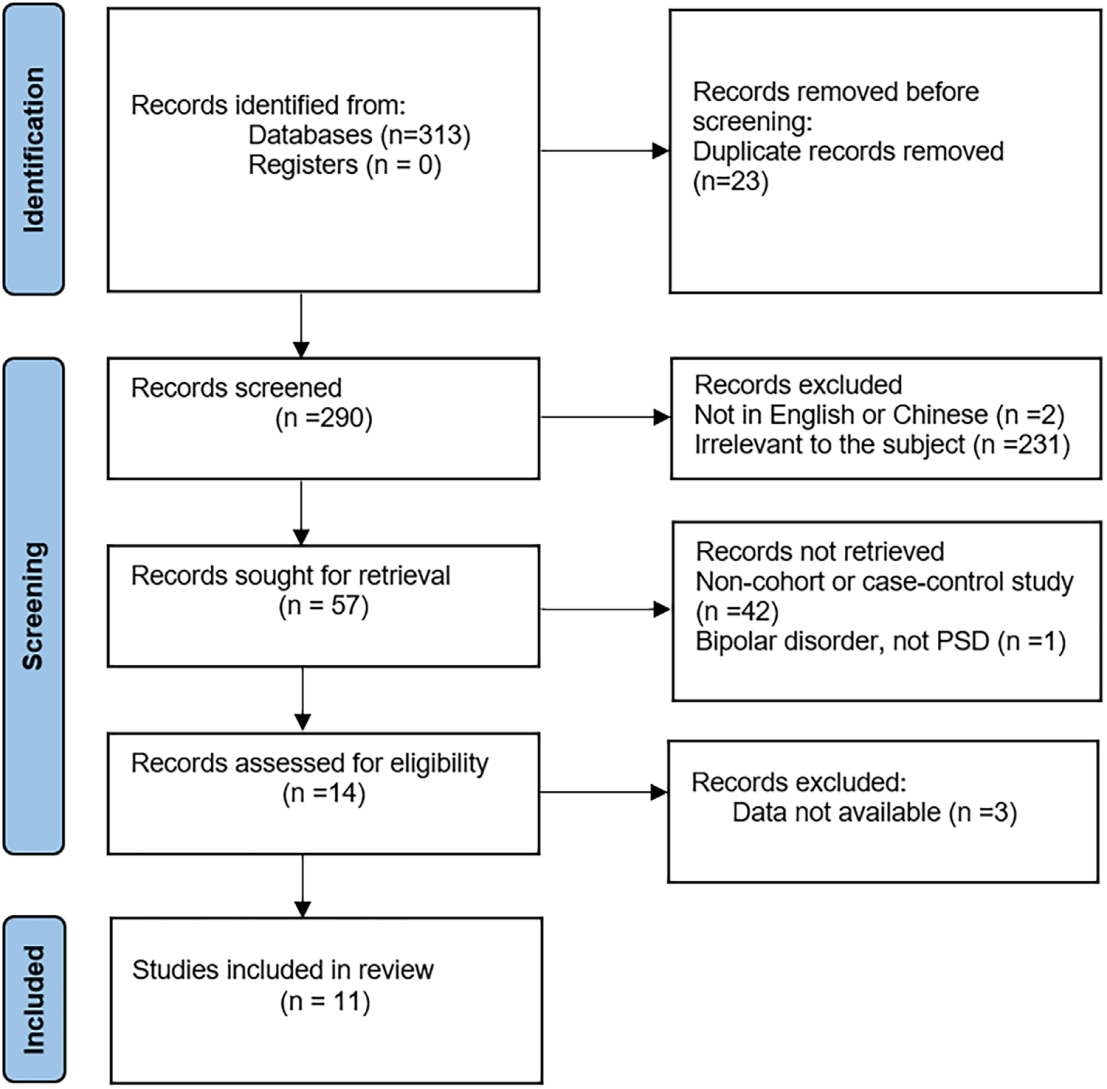
Flowchart of study search and selection

A total of 313 related articles were obtained through the search strategy. Twenty‐three duplicates were removed by endnote first. Two hundred and seventy‐six articles were excluded by preliminary screening the titles or abstracts; 3 of which were deleted in strict accordance with the inclusion and exclusion criteria after reviewing the remaining articles and 11 studies were considered eligible after a detailed review (Figure 1).

### 3.2 Characteristics and quality assessment of included studies

This process resulted in selection of 11 studies involving 2789 participants, of whom 842 were PSD patients, for the meta‐analysis (Table 1). Of the included studies, first author, publication time, sample size, country of origin, diagnosis of stroke and PSD, time points when PSD was assessed, Hcy levels (mean ± standard), and other elements were listed. This study analyzed the diagnosis time of PSD, year of publication, and stroke type, and compared the Hcy levels of PSD patients and non‐PSD patients in acute stroke from different aspects. The studies included in the meta‐analysis were generally of high quality (Table 1).

**TABLE 1:** Characteristics of studies included

											Number		Hcy level (mean ± SD)				
Author	Year	Country	Type of stroke	Admission time	Stroke assessment	Severity assessment	Type	Laboratory findings	Depression assessment	Time	PSD	non‐PSD	PSD	non‐PSD	Outcomes	Study design	NOS
Zhao	2020	China	First‐ever AIS	NA	MRI/CT	NIHSS	TOAST	HCY, FBG, hs‐CRP	DSM‐IV (HMD‐17)	3	55	181	18.17 ± 7.31	15.17 ± 5.38	PSD, mRS	Cohort study	7
Lu	2019	China	First‐ever AIS	48 h	MRI/CT	NIHSS	TOAST	HCY, CRP, FBG, IL‐6	BDI‐FS	3	76	234	18.17 ± 6.12	14.70 ± 4.77	PSD, mRS	Cohort study	8
Yin	2018	China	AIS	48 h	MRI/CT	NIHSS	Lacunar/thrombotic	HCY, hs‐CRP	DSM‐IV (HMD‐24)	3	241	357	16.10 ± 7.83	15.93 ± 8.19	PSD	Cohort study	6
Li	2017	China	First‐ever AIS	24 h	MRI/CT	NIHSS	TOAST	HCY, FBG, hs‐CRP, WBC	DSM‐IV (HMD‐17)	3	65	173	20.43 ± 5.23	24.60 ± 5.46	PSD	Cohort study	7
Tang	2016	China	First‐ever AIS	24 h	MRI/CT	NIHSS	TOAST	HCY, FBG, hs‐CRP, WBC	DSM‐IV (HMD‐17)	6	69	157	17.77 ± 8.25	15.20 ± 4.94	PSD, mRS, ACM	Cohort study	7
Zhu	2016	China	AIS	7 days	WHO‐MONICA	NIHSS	TOAST	HCY, hs‐CRP, ferritin	DSM‐IV (HMD‐17)	2	56	140	7.58 ± 5.52	7.97 ± 5.30	PSD, BI, mRS MMSE	Cohort study	6
Yang	2015	China	AIS	24 h	MRI/CT	NIHSS	TOAST	HCY, hs‐CRP, ferritin	DSM‐IV(HMD‐17)	6	69	159	17.73 ± 6.51	13.27 ± 5.31	PSD, mRS, ACM	Cohort study	6
Cheng	2014	China	First‐ever AIS	24 h	WHO‐MONICA	NIHSS	TOAST	HCY, hs‐CRP, GPT, GOT, FPG, glutamate	DSM‐IV (HMD‐17)	3	70	139	17.13 ± 6.21	12.73 ± 5.09	PSD, mRS, ACM	Cohort study	7
Li	2014	China	First‐ever AIS	24 h	MRI/CT	NIHSS	TOAST	HCY, hs‐CRP,	DSM‐III‐R	3	44	147	15.46 ± 15.17	14.83 ± 5.32	PSD	Cohort study	7
Yue	2014	China	First‐ever AIS	24 h	MRI/CT	NIHSS	TOAST	HCY, FBG, hs‐CRP, WBC, 25[OH] D	DSM‐III‐R	6	60	184	19.63 ± 9.49	15.20 ± 5.83	PSD, mRS	Cohort study	6
Kausik Chatterjee	2010	England	Stroke	NA	WHO‐MONICA	NA	NA	HCY, uric acid, cholesterol, LDL‐C, HDL‐C, triglyceride, folic acid	DSM‐IV (MADRS>17)	9	37	76	16.6 ± 3.32	14.23 ± 1.74	PSD, BI, mRS MMSE, FAI	Case control study	6

AIS, acute ischemic stroke; FAI, the 66‐point Frenchay Activities Index; FBG, fasting blood glucose; FPG, fasting plasma glucose; GOT, glutamate oxaloacetate transaminase; GPT, glutamate‐pyruvate transaminase; HCY, homocysteine; Hcy level, using μmol/L as unit; hs‐CRP, hypersensitivity C‐reactive protein; Hs‐CRP, high‐sensitivity C‐reactive protein; mRS, modified ranking scale; MMSE, Mini‐Mental State Examination; NIHSS, National Institutes of Health Stroke Scale; NOS, Newcastle–Ottawa Scale; PSD, poststroke depression; SD, standard deviation; Time, time points of depression assessment (month); 25[OH] D, 25‐hydroxyvitamin D.

### 3.3 Hcy level in the acute phase of stroke and PSD

#### 3.3.1 Meta‐analysis

**FIGURE 2:**
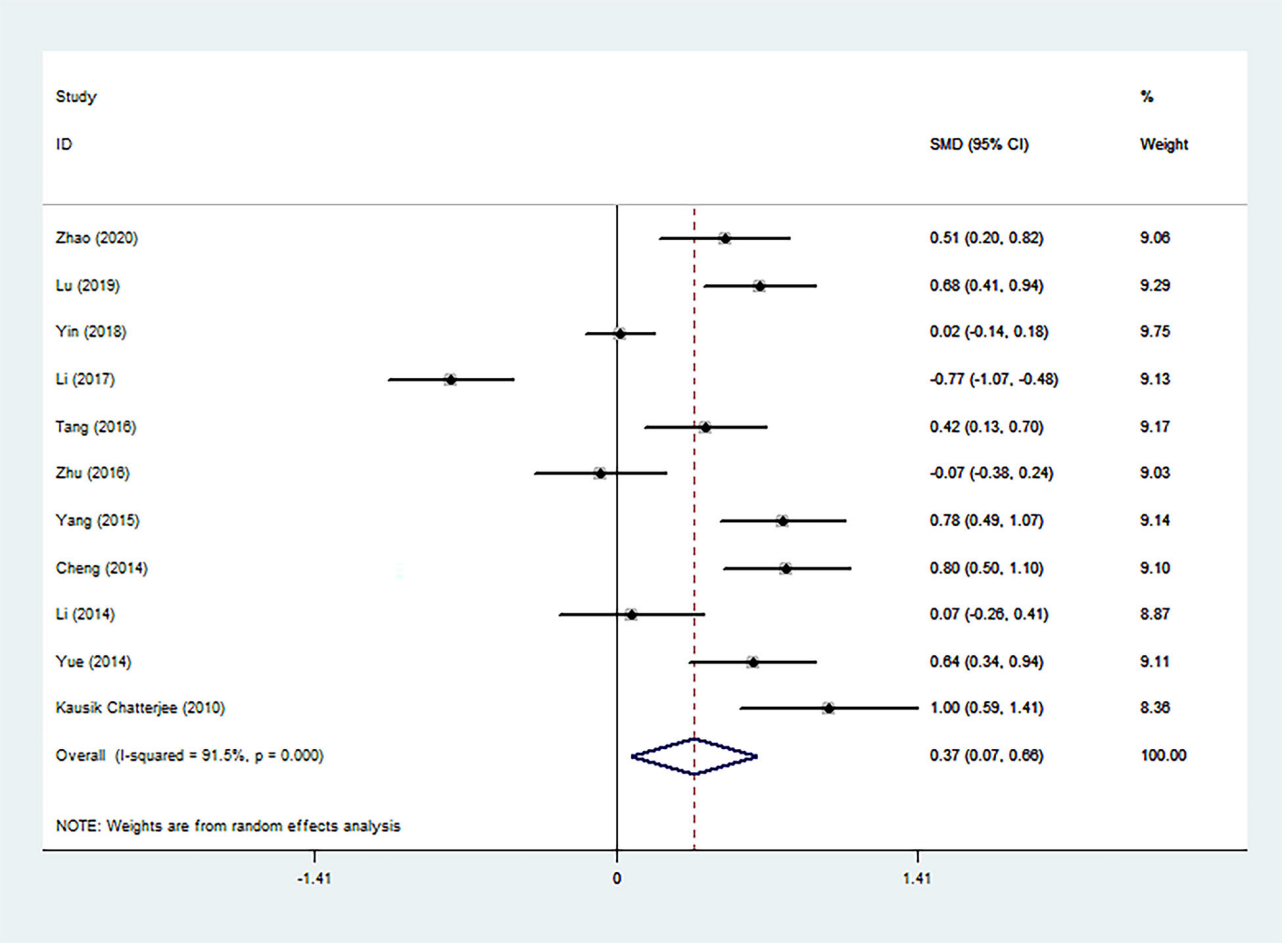
Forest plot of Hcy level in PSD patients and the non‐PSD

There was obvious heterogeneity among the studies reporting differences of Hcy levels between PSD patients and non‐PSD patients (*I* ^2 ^= 91.5%). Thus, a random effects model was used to pool the data. An incorporate analysis showed that the PSD patients had significantly higher levels of Hcy compared to the controls (SMD = 0.37, 95% CI = 0.07–0.66, *p *< .001) (Figure 2).

#### 3.3.2 Sensitivity analysis

**FIGURE 3:**
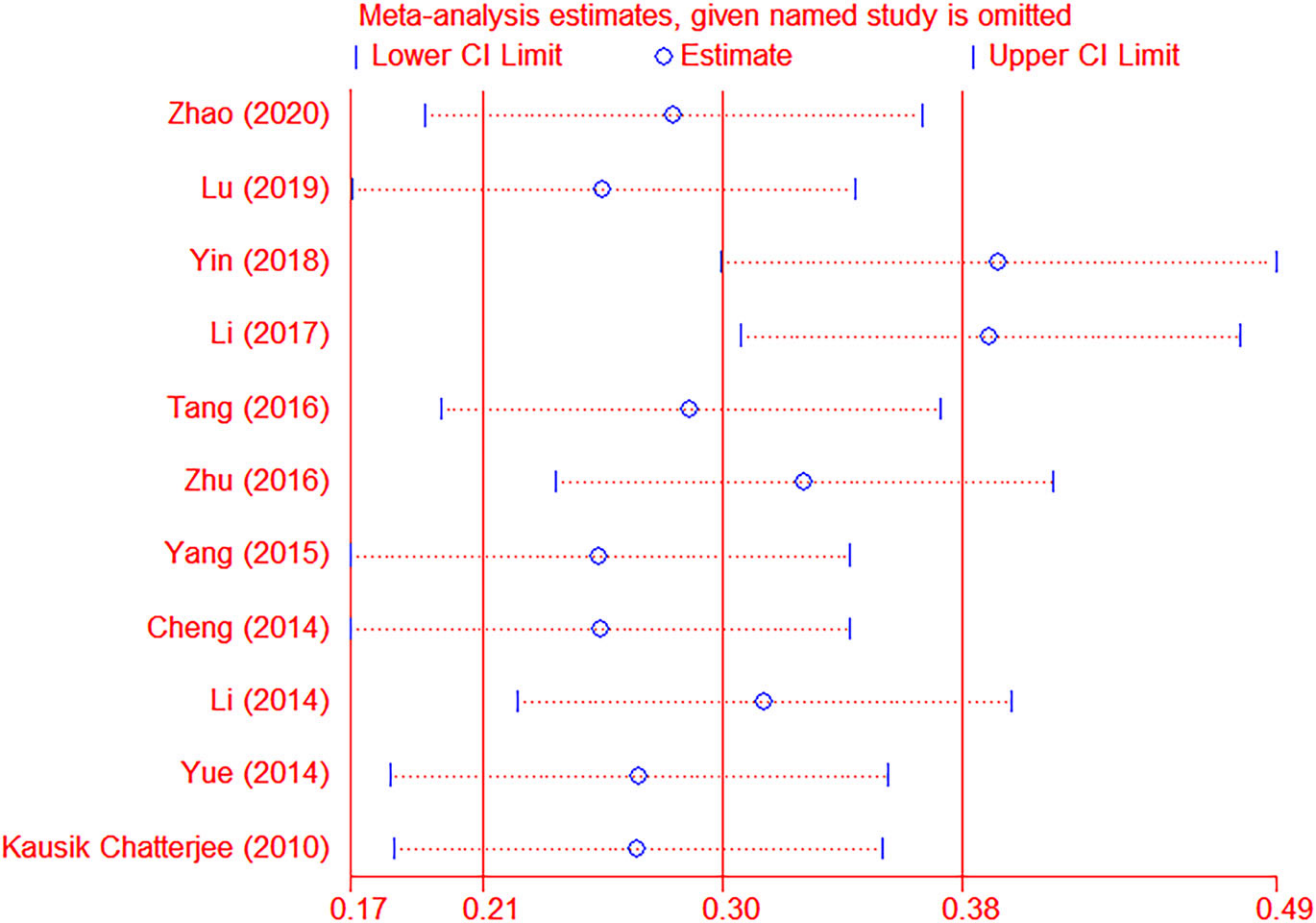
Sensitivity analysis chart

Sensitivity analysis evaluated the impact of each study on the amount of combined effects through different statistical methods and determined whether any studies increased heterogeneity due to significant data differences. The results showed that two articles were the sources of heterogeneity in this meta‐analysis (Figure 3).

#### 3.3.3 Subgroup analysis

**FIGURE 4:**
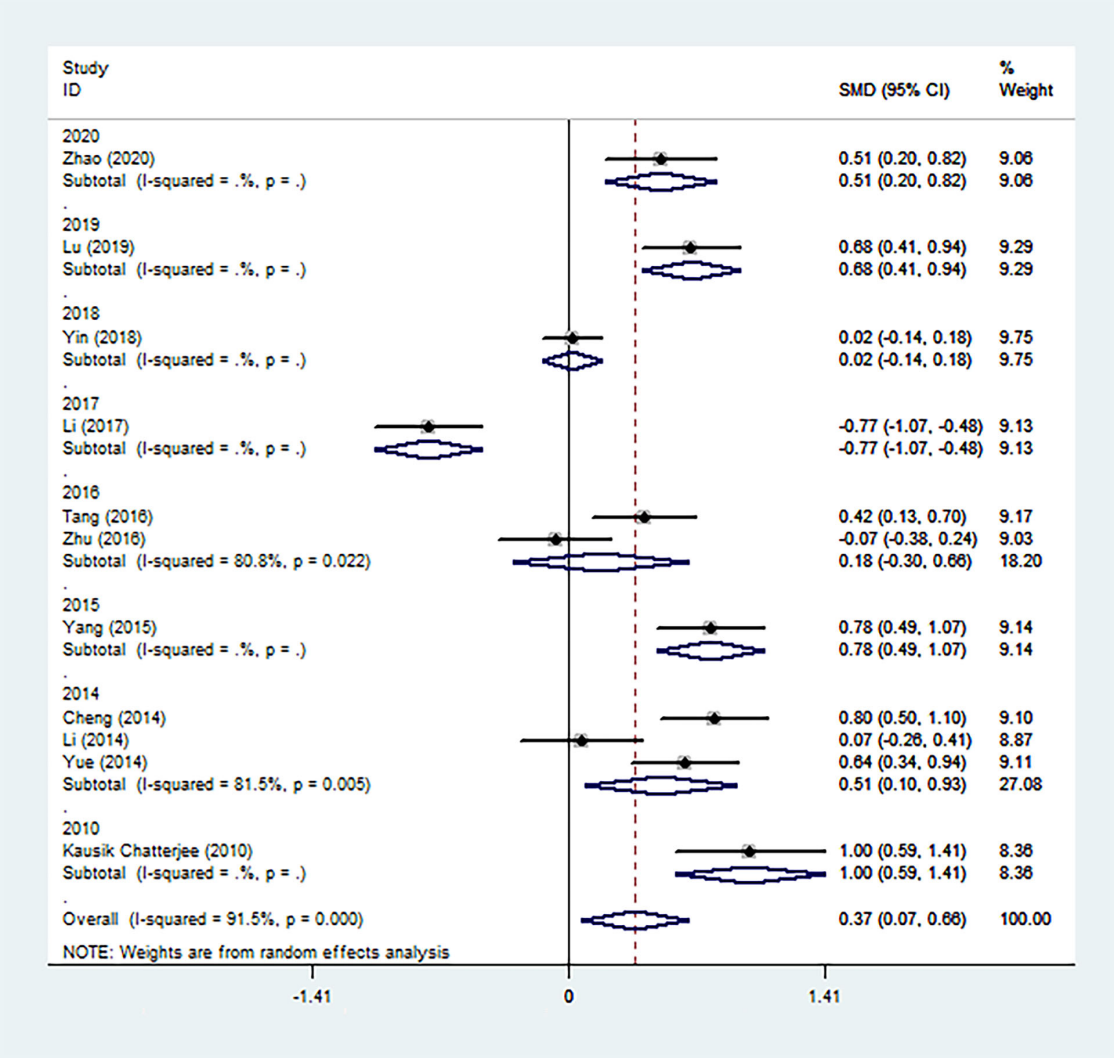
The effects of publication time on Hcy were compared in the random effect model (2020, 2019, 2018, 2017, etc. represent the year of publication)

**FIGURE 5:**
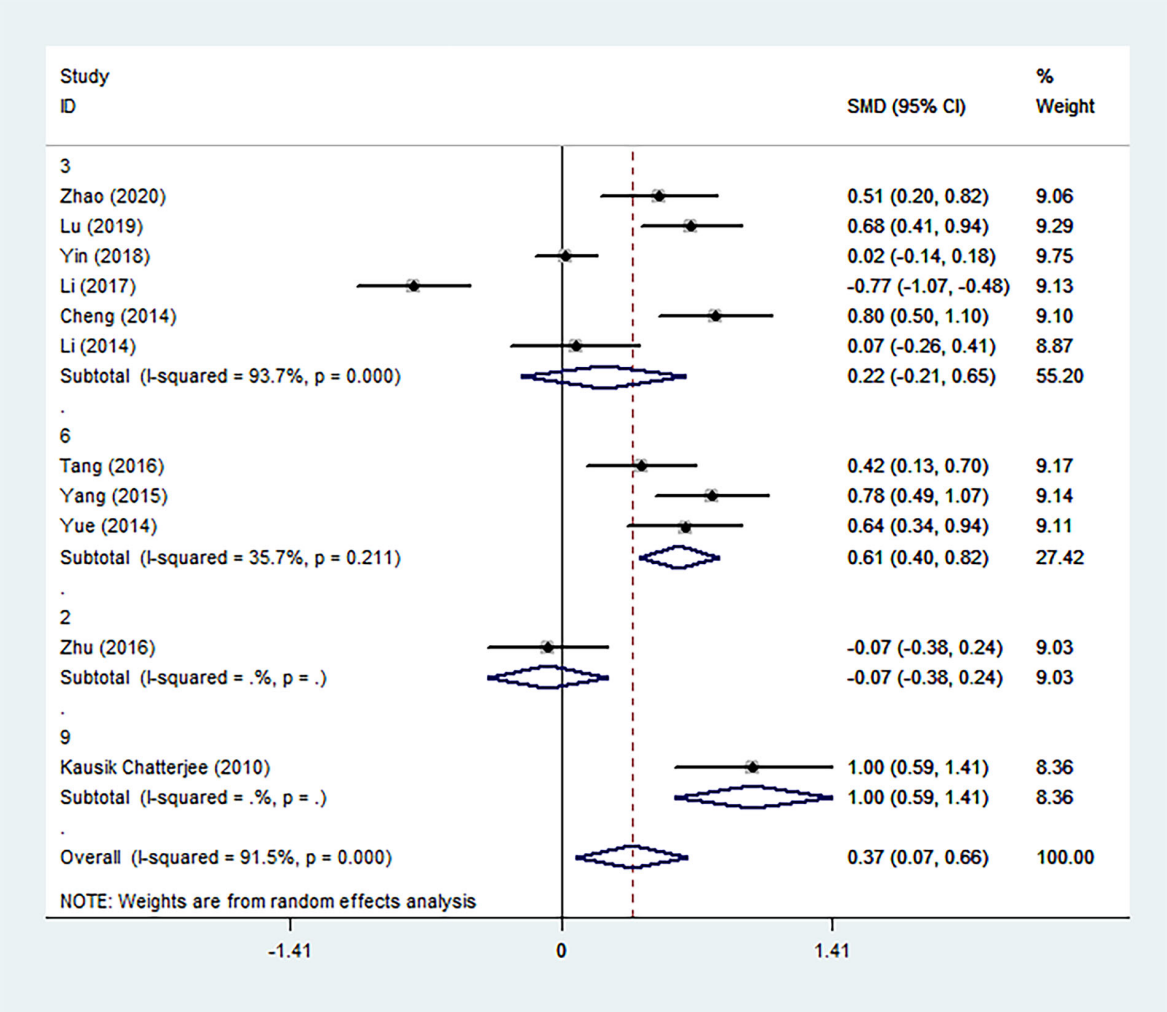
The effects of the time of diagnosis of PSD on Hcy were compared in the random effect model (3, 6, 2, 9 represent the time to diagnosis of PSD at 3, 6, 2, 9 months after stroke)

**FIGURE 6:**
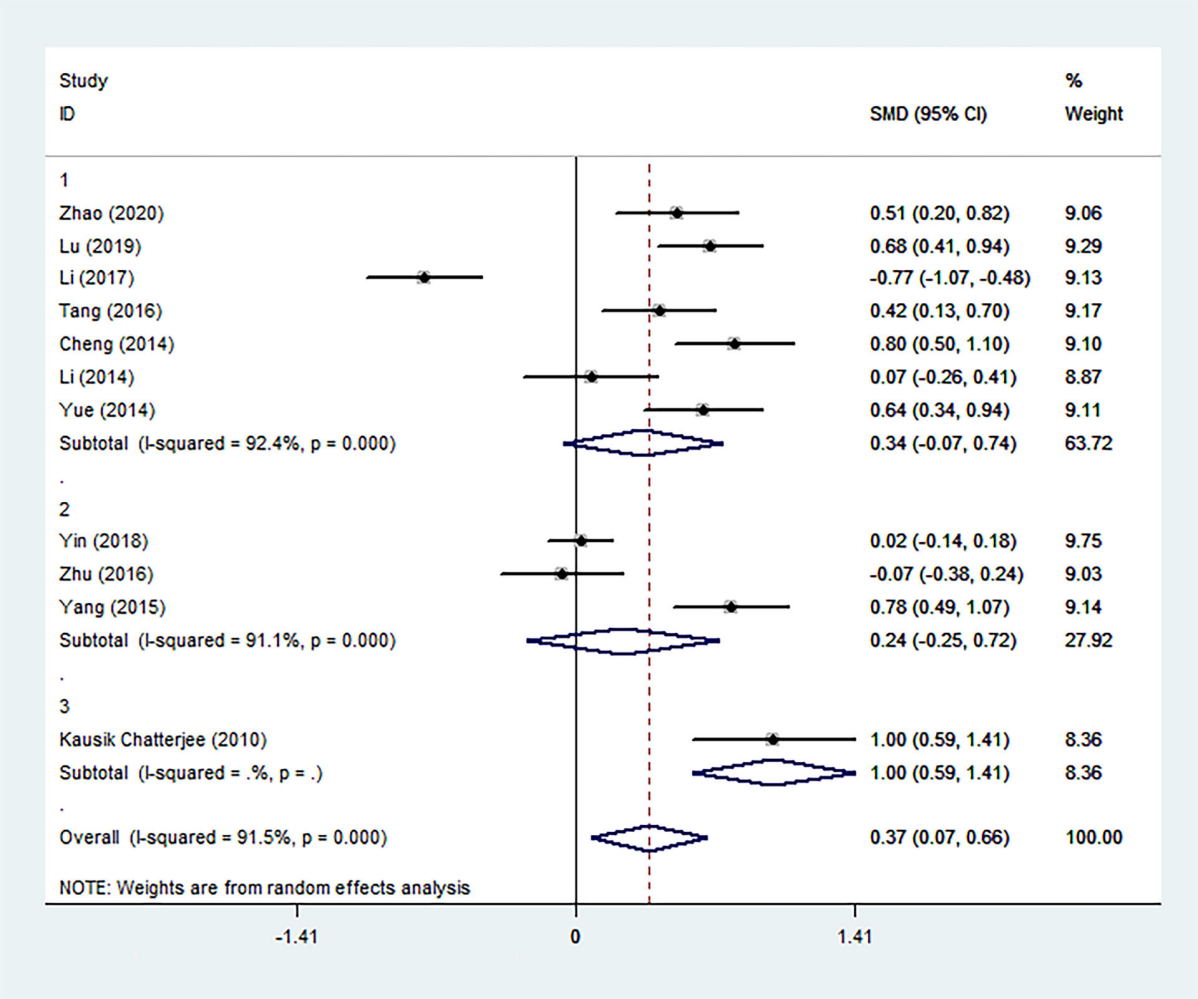
The effects of patients with different inclusion criteria on Hcy were compared in the random effect model (1, the incluson criteria for patients were first‐ever AIS; 2, the incluson criteria for patients were AIS; 3, the incluson criteria for patients were stroke only)

We performed three subgroup analyses of the included studies based on clinical and methodological differences, and the results were collected and shown (Figures 4, 5, 6). In most subgroup analyses that included more than one study, the conclusion remained that patients with PSD had higher Hcy levels during the acute stroke period than patients without depressive symptoms. It is worth noting that there is a significant difference in the levels of Hcy in the acute phase between survivors diagnosed with PSD more than 3 months after stroke and non‐PSD survivors (6 months: SMD = 0.61, 95% CI: 0.40– 0.82; 9 months: SMD = 1.00, 95% CI: 0.59– 1.41), but it does not exist within 3 months (2 months: SMD = −0.07, 95% CI: −0.38– 0.24; 3 months: SMD = 0.22, 95% CI: −0.21– 0.65). Through subgroup analysis of publication time, it can also be concluded that the main heterogeneity comes from the following three articles. These three articles are the research published by Zhu et al. in 2016 (SMD = −0.07, 95% CI: −0.38– 0.24), Li et al. in 2017 (SMD = −0.77, 95% CI: −1.07– −0.48), and Yin et al. in 2018 (SMD = 0.02, 95% CI: −0.14– 0.18).

### 3.4 Publication bias

**FIGURE 7:**
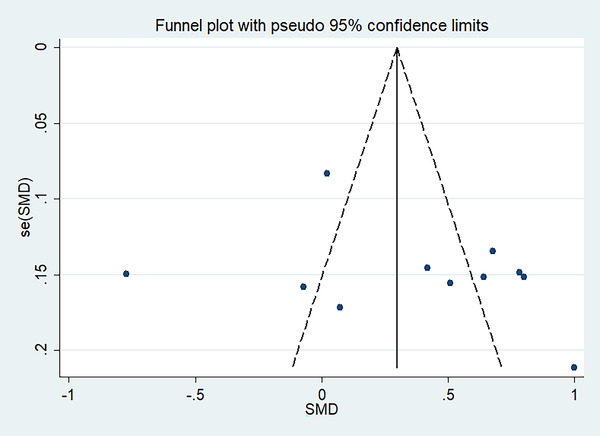
The funnel plot for the meta‐analysis

The funnel plot and Egger test were used to quantitatively evaluate the publication bias of literature on stroke. The results of Egger test provided statistical evidence for the symmetry of funnel chart in the overall results (*p* = .286), indicating that there was no obvious publication bias (Figure 7).

## 4 DISCUSSION

### 4.1 Interpretation of findings

The meta‐analysis included 11 studies with 2789 participants to reveal that acute stroke patients with higher Hcy levels had higher risks of PSD. The incidence of PSD in this study was 30.19%, which was similar to the results of previous studies. Original conclusions remained after removing studies with larger heterogeneity ratios. Stroke type also had no effect on outcomes. In addition to this, three articles (Cheng et al., 2018; Pascoe et al., 2012; Xu et al., 2018) for which original data were not available also demonstrated that elevated Hcy levels in the acute phase of stroke are significantly associated with PSD.

Hcy is the intermediate product of methionine cycle. It can produce sulfide with the participation of vitamin B6 and reduce to methionine under the action of vitamin B12. The increase of Hcy level can consume vitamin B12, which is a coenzyme of 5‐hydroxytryptamine (5‐HT) metabolism (Coppen & Bolander‐Gouaille, 2005). Meanwhile, 5‐HT reduction has been proved to be one of the pathogenesis of depression (Daut & Fonken, 2019; Zhang et al., 2014), which indirectly suggests that the amelioration of depression may be achieved by lowering Hcy. Hcy can injure tissue endothelial cells, make platelet adhesion and aggregation, homogenization of smooth muscle cells, and affect thrombin regulatory protein activity by inhibiting the binding of endothelial cells and tissue‐type plasminogen activator. In addition, Hcy can activate the apoptosis program in nerve cells, inhibit cell membrane sodium potassium enzyme and induce neuronal metabolic dysfunction (Folstein et al., 2007; Lipton et al., 1997; Mattson & Shea, 2003). To a certain extent, Hcy promotes the occurrence of acute ischemic stroke. So whether in stroke or depression, Hcy seems to play an important role. Hcy, as a common serological indicator, has also attracted much attention for its association with PSD. Zhu et al. (2016) showed that the median Hcy level was 8.00 μmol/L in PSD patients but 9.00 μmol/L in non‐PSD patients 2 months after acute stroke. Pascoe et al. (2012) found that Hcy was significantly associated with depressive symptomatology in elderly Swedish stroke survivors. In addition, Xu et al. (2018) reported that Hcy was a significant predictor of poststroke depression.

To our knowledge, this meta‐analysis is the first study to focus on the correlation with Hcy levels and the risk of PSD at admission in stroke survivors. Our findings confirmed that higher Hcy level in the acute phase of stroke is associated with a higher risk of PSD. Although the value of *I* ^2^ of this analysis was 91.5%, the degree of heterogeneity was high. However, we identified the source of heterogeneity by subgroup analysis and sensitivity analysis. The heterogeneity was mainly derived from the time of diagnosis of PSD, so more clinical investigations with uniform time of diagnosis may be needed in the future. However, each of the studies included in this analysis received a high NOS score and therefore the data remain reliable.

### 4.2 Clinical implications

PSD is the most common noncognitive neuropsychiatric complication after stroke. The findings of this meta‐analysis showed that Hcy, as the most common serological index, was an independent predictor of PSD. This showed that high Hcy level suggested a worse prognosis for stroke survivors. Fortunately, high Hcy can return to normal by changing lifestyle, such as eating more folate rich food, quitting alcohol, quitting smoking, etc. It is suggested that in the future, the level of Hcy in acute stroke should be paid more attention and intervened in time by medical staff.

### 4.3 Limitations

It must be admitted that there are some limitations in this meta‐analysis. First, the sample size of the included studies is small, and all but one of them are from China, so we should be cautious when extending these results to other countries. Second, this study has language limitations, so some studies in non‐English speaking countries are not included. Third, there is great heterogeneity among the included studies, which may threaten the internal validity of meta‐analysis results. More studies are needed in the future to determine the effect of Hcy on PSD.

## 5 CONCLUSION

This meta‐analysis found that the Hcy of PSD survivors in the acute phase of stroke was significantly higher than that of non‐PSD survivors; that is, the higher the levels of Hcy in the acute phase of stroke, the higher the risk of PSD, whether it was the first stroke or not. Therefore, Hcy is an important potential predictor of PSD.

## CONFLICT OF INTEREST

Authors have no conflict of interest to declare.

## AUTHOR CONTRIBUTIONS

CYP is first author. CYP and ZH designed the study. CYP and ZH collected the data. PMD and CY were involved in data cleaning. CYP and ZH analyzed the data. CYP drafted the manuscript. CYP and ZH checked the data. All authors have read and approved the final manuscript.

### PEER REVIEW

The peer review history for this article is available at https://publons.com/publon/10.1002/brb3.2626.

## Data Availability

The data used to support the findings of this study are included within the article.
